# Ceviche-Natural Preservative: Possibility of Microbiota Survival and Effect on *L. monocytogenes*

**DOI:** 10.3390/foods11060860

**Published:** 2022-03-18

**Authors:** Arkadiusz Józef Zakrzewski, Wioleta Chajęcka-Wierzchowska, Anna Zadernowska

**Affiliations:** Department of Industrial and Food Microbiology, University of Warmia and Mazury, Plac Cieszyński 1, 10-726 Olsztyn, Poland; arkadiusz.zakrzewski@uwm.edu.pl (A.J.Z.); wioleta.chajecka@uwm.edu.pl (W.C.-W.)

**Keywords:** ceviche, *Listeria*, *Listeria monocytogenes*, survival

## Abstract

Ceviche is a marinated raw fish dish ready for consumption; it is a part of the cuisine of various countries on the Pacific coast and its preparation may differ among them. Although the process uses the traditional method of food preservation by lowering the pH, the exposure time is very limited, so the aim of the study was to determine the viability of bacteria often isolated from fish after the process of preparing traditional ceviche. For this purpose, the traditional plate method and flow cytometry were used, and for pathogenic *L. monocytogenes* strains, the influence of stress during the preparation of the dish on the pathogenic potential was determined. The study showed that the highest percentage of viable cells was observed in the case of *L. monocytogenes* and remained at the level of 98.54%, slightly less for *L. innocua*, 96.93%. For the remaining species the reduction did not exceed 10%, for *E.* *faecalis* it was 92.76%, for *S. liqefaciens* 91.44%, *H. alvei* 93.68%. In addition, the study of the antibacterial properties of individual ingredients showed that habanero and coriander did not show any bactericidal effect, while for onions the amount of live cells was 99.11%, and for lime juice 97.26%, Additionally, the study of changes in virulence, antibiotic resistance and gene expression showed that the stress during the preparation of ceviche has different effects depending on the strain and may cause virulence potential increase, levofloxacin and daptomycin minimum inhibiotory concentration increase and some crucial virulence gene expression induction; therefore, it is important to take care of the quality of the products used to prepare the ceviche and accurate pretreatment.

## 1. Introduction

Ceviche (or cebiche) is a traditional dish in many Latin American countries; however, it is particularly popular in Peru and Mexico, though, over the years, growing popularity of this dish in restaurants and special events in countries outside Latin America can be observed. Traditionally, it is made from raw fish meat that is marinated in citrus juices for up to 30 min to kill potential pathogens and give the meat a taste and texture similar to cooking. Lime juice (*Citrus aurantifolia*) is one of the most widely used ingredients in its production, regardless of the region in which it is produced. However, depending on where it is made, other citrus juices such as sour orange and weak organic acids such as acetic acid can also be used. These products have been described as effective antibacterial agents due to their dissociation within the bacterial cell into a proton, which ultimately reduces the pH of the cytoplasm, and an anion, which induces metabolic disorders in the acidified cytoplasm [1]. Lime juice has shown antibacterial activity against *Vibrio parahemolyticus* and *Vibrio cholerae* when used on raw shellfish [2,3]. However, the marinating time is important, as the ceviche preparation takes only up to 30 min, which may not be enough time to reduce pathogens and food spoilage bacteria. The pilot studies conducted for the project focused on the food spoilage bacteria order Enterobacterales and showed that the most common genera were *Serratia* spp. and *Hafnia* spp., so they were used in this study. Additionally, in non-Latin American countries, one of the main threats isolated from fish and seafood is *L. monocytogenes*, which is the causative agent of listeriosis, a serious foodborne illness with a high associated case-fatality rate [4], so the aim of the study was a determination survival of bacteria including food spoilage bacteria; *Serratia* spp. and *H. alvei*, opportunistic pathogens; *Enteroccocus faecalis* and pathogenic *L. monocytogenes* isolated from fish meat using a sensitive method of flow cytometry and the effect that the stress of ceviche preparation may have on *L. monocytogenes*.

## 2. Materials and Methods

### 2.1. Bacterial Strains and Culture Methods

Strains isolated from salmon (*Salmo salar*) and trout (*Oncorhynchus mykiss*) were used in the survival study, including the following: 5 strains belonging to the species *Listeria. monocytogenes*, 5 strains belonging to the species *Listeria innocua*, 5 strains belonging to the species *H. alvei*, 5 strains belonging to the species *Seratia liquefaciens* and 5 strains belonging to the to the species *E.s faecalis* (Table S1). Additionally, twenty *L. monocytogenes* strains were used in the study of changes in the antibiogram in order to increase the number of biological samples (Table S2). All strains used in the study come from the strain collection of the Department of Industrial and Food Microbiology of the University of Warmia and Mazury in Olsztyn. The strains were kept in microbanks (Biomaxima, Lublin, Poland) at −80 °C until use. Tryptic soy broth (TSB; Merck, Darmstadt, Germany) was used to prepare 24 h bacterial cultures. Aliquots (2 mL) from each overnight culture were added to fresh TSB tubes and incubated for 24 h at 37 °C.

### 2.2. Ceviche

A ceviche recipe was prepared as follows 680 g of mahi-mahi (*Coryphaena hippurus*) with coriander (*Coriandrum sativum*) (30.8 g), red onion (*Allium cepa*) (50 g), habanero pepper (*Capsicum chinense*) (6.2 g), marinated together with lime juice (*Citrus aurantifolia*) (236 g) for 30 min.

### 2.3. A Limited Medium—Fish Flesh Medium

From the need to use a clear medium in cytometric analyses, the medium proposed by Chandrasekaran (1985) [5], with modifications, was prepared. Fresh mahi-mahi (*Coryphaena hippurus*) fish fillets were washed with distilled water and the meat was then cut into small pieces. One hundred Grams of meat was homogenized with an equal volume of tap water, and another 100 g with 1% NaCl solution. Homogenates were prepared in a volume of 1000 mL and centrifuged (10,000 rpm for 30 min at room temperature (22 ± 2 °C)). The supernatant was mechanically filtered using filters 0.2 μm and stored at refrigeration temperature.

### 2.4. Flow Cytometry

#### 2.4.1. Flow Cytometer Adjustment

Fluorochrome stock solutions were prepared with accordance to producer instructions by adding SYTO9 (5 mM) and PI (20 mM) in proportion 1:1 to a tube as a working solution. For live (LCS) and dead (DCS) cell suspensions, 1 mL of 24 h bacterial suspension was centrifuged at 10,000× *g* for 5 min and resuspended in 1 mL of PBS for LCS or 1 mL of 70% isopropanol for DCS. Staining was performed by adding 2 mL of LCS or DCS with 6 µL of working solutions. The mixtures were incubated for 15 min at room temperature and read with a flow cytometer (FACSlyric, Beckton Dickinson, Franklin Lakes, NJ, USA). The excitation laser of the flow cytometer had a wavelength of 488 nm and the fluorescence emissions were collected in the red and green channels. SYTO9-stained LCS and PI-stained DCS were used to locate appropriate bacterial populations and standardize the most appropriate cytometric parameters to avoid interference from emission spectra. Forward scatter, side scatter, and fluorescence were recorded with logarithmic signal amplification. First, the forward and side scatter enhancements were configured, followed by the green fluorescence enhancement to detect live bacteria, and finally the red fluorescence enhancement was combined to indicate dead bacteria.

#### 2.4.2. Antibacterial Properties of Ceviche and Ceviche Components

To evaluate antimicrobial properties of ceviche and ceviche components fish flesh medium were used. To use the same proportion as for traditional ceviche, the amount of fish media used were equal to fresh fish ceviche, achieving a pH of 3.2–3.3. Each of the ceviche ingredients was separately combined with the fish flesh media, then homogenized, and filtered with a vacuum pump on 0.2 µm pore size filters. The filtered medium was combined with the previously centrifuged bacterial cultures so that the final concentration was about 6 × 10^8^ cfu/mL. The samples were incubated at 4 °C for 30 min, then the samples were centrifuged for 10 min at 10,000× *g*, the supernatant was removed, and 1 mL of PBS was added. Then, steps from the flow cytometer adjustment section were followed.

### 2.5. Bacterial Total Count

Samples (10 g) of ceviche (time = 0 and time = 30 min) were aseptically taken and homogenized in stomacher (Masticator Homogenizator, IUL S.A., Barcelona, Spain) with 90 mL NaCl solution (9 g/L). The homogenate was serially diluted, and the total viable rate (TVC) was determined by the pore plate method using Nutrient Agar (Merck, Darmstadt, Germany). The plates were incubated at 30 °C for 72 h and cfu/g were determinate. Additionally, determination of dominant morphological colonies was performed. For this purpose, the 10 most frequent colonies (based on morphological characteristic) were selected and then identified using the MALDI-TOF system. Measurements were taken using a VITEK^®^ MS (bioMérieux, Marcy l’Etoile, France) with acceleration voltage 200 kV, mass range 2–20 kDa, laser frequency 50 Hz, and extraction delay time 200 ns. All mass fingerprints were analyzed by the VITEK^®^ MS v2.0 MALDI-TOF mass spectrometry systemV2.0 research use only (RUO; SARAMIS version 4.13) databases (bioMérieux, Marcy l’Etoile, France).

Isolates were tested in duplicate using the direct transfer protocol according to the manufacturers’ recommendations. Briefly, the isolates were cultured for 48 h at 30 °C on TSA (Merck, Darmstadt, Germany) than were transferred to the target plate. One microliter of MALDI matrix VitekMS-CHCA (bioMérieux, Marcy l’Etoile, France) (In the case of identification of yeasts, this step was preceded by the addition of 0.5 µL of formic acid) was added to the spots. After crystallization of the matrix solution, the target was loaded into the MALDI-TOF MS, and the analysis was started. The confidence level was determined in percentage.

### 2.6. Phenotypic and Genotypic Changes

#### 2.6.1. Virulence Potential Changes in a Live Model

*Galleria mellonella* larvae were infected with *Listeria monocytogenes* (~100 CFU/larvae). The studies were carried out on strains that had previously been subjected to stress related to ceviche preparation. Bacterial inoculums were injected dorsolaterally into the hemocoel of last-instar larvae using a 10 μL disposable Hamilton syringe. After injection, the larvae were incubated at 37 °C. Larvae were considered dead if they did not move when touched. Galleria larvae infused with 0.9% NaCl were a control group [6].

#### 2.6.2. RNA Isolation and Reverse Transcription into cDNA

Total RNA was isolated from the overnight culture (control) and strains subjected to the ceviche preparation process using the Total RNA Mini Plus Kit (A&A Biotechnology, Gdynia, Poland) and purified and concentrated according to the manufacturer’s instructions using the CleanUp RNA Concentrator (A&A Biotechnology, Gdynia, Poland). RNA integrity of all samples was checked by loading 10 µL RNA into a 1.2% agarose gel in 0.5% TBE buffer and running at 90 V for 1 h. Once the RNA integrity is confirmed by visualizing the two bands (16S and 23S RNA) with little smearing. RNA concentration and purity were measured optically using a DeNovix DS11 FX spectrophotometer/fluorometer (DeNovix Inc., Wilmington, NC, USA) based on sample absorption at wavelengths of 260 nm and 280 nm. All RNA samples were immediately partially reverse-transcribed and the rest stored at −80 °C. All the RNA samples were normalized to 5 µg/µL and transcribed into the cDNA using the TranScriba kit (A&A Biotechnology, Gdynia, Poland) for the synthesis of first-strand cDNA. This kit uses recombinant MMLV reverse transcriptase, which has low RNAseH activity at 37–42 °C and optimal DNA polymerase activity. Template RNA was protected with a recombinant RNAse inhibitor. Random sequence hexamer was used as a primer.

#### 2.6.3. Real Time PCR Analysis of Gene Expression

Nine genes were selected for expression evaluation: *cspL*A, *gad*B, *opu*CA*, rpo*E, *fla*A, *inl*A, *mpr*F, *lin* and *fos*X. For each gene, a threshold line and quantitative cycle (Cq) were determined using the Rotor-Gene series software (Qiagen Inc., Montreal, ON, Canada). Genomic DNA standard curves (5 points of dilutions. For each primer pair, amplification efficiency was calculated as E = 10 (−1/slope) [7,8]. The level of gene expression was determined fluorometrically using SYBR green with the RotorGene Q system (Qiagen Inc., Montreal, ON, Canada). Samples were examined for differences in gene expression using relative quantification, in which relevant gene expression is normalized to a housekeeping gene using the mathematical model proposed and reviewed by [7]. A threshold was determined by the software for real-time PCR reactions. Expression ratios equal to or greater than 2 represented a significant increase in gene expression, while expression ratios equal to or less than 0.5 represented a significant decrease in gene expression. Primer sequences for the housekeeping gene 23 S rRNA as well as the virulence-associated genes *gel*E, *asa*1, *esp* and *cyl*L were obtained from Shepard and Gilmore (2002) (Table S3). Gene expression analysis was performed three times. Additionally, three independent real-time PCR reactions were conducted in each replicate. The RGEs detected for each gene and each stress condition were considered significant (*p* < 0.05) on the basis a t-test performed using the RGE of the control as reference.

Real-time PCR reactions was performed in a final volume of 20 μL containing 10 μL of PowerUp SYBR Green MasterMix (ThermoFischer Scientific, Waltham, MA, USA), 1.0 μL of each primer (10 pmol/1 μL) and 5 ng of template cDNA, filled up with ddH_2_O to a final volume. The real-time PCR cycling conditions were following 50 °C for 2 min, 95 °C for 2 min and 40 cycles of 15 s at 95 °C, 15 s at temperature adjust for primer set and 60 s at 72 °C. The specificity of the real-time PCR product was checked by the construction of the melting curve by heating in a slow ramp between 60 and 95 °C in increments of 0.5 °C for 5 s.

#### 2.6.4. Antibiotic Susceptibility

The study of changes in susceptibility to antibiotics was carried out on strains from 24 h culture (control) and those subjected to stress related to the preparation of ceviche. VITEK 2 Compact with AST–P643 (bioMérieux, Marcy l’Etoile, France) was used to access the antimicrobial susceptibility of the A8-1 to 18 clinical antibiotics, which included benzylpenicillin (Bp, 0.125–64 μg/mL), ampicillin (Amp, 0.25–32 μg/mL), high-level gentamicin (synergistic) (HLG, 500 μg/mL; Gen, 8–64 μg/mL), high-level streptomycin (HLS, 1000 μg/mL), clindamycin (Cli, 0.125–4μg/mL), levofloxacin (Lev, 0.12–8 μg/mL), erythromycin (E, 0.25–8 μg/mL), quinupristin/dalofopine (Qd, 0.25–16 μg/mL), linezolid (Lzd, 0.5–8 μg/mL), vancomycin (VA, 0.5–32 μg/mL), tetracycline (Tet, 1–16 μg/mL), tigecycline (Tgc, 0.12–2 μg/mL), chloramphenicol (C, 4–64 μg/mL), daptomycin (Dap, 0.12–8 μg/mL), Trimethoprim/sulphamethoxazole (SXT, 0.5/9.5–16/304 μg/mL), and nitrofurantoin (F, 16–512 μg/mL). According to the MIC obtained, the results were judged according to Clinical Laboratory Standard Institute criteria (CLSI M100) (Clinical and Laboratory Standards Institute, 2018) and the European Food Safety Authority (EFSA) for assessment of bacterial resistance to antimicrobials. The study increased the bioassays to 20 to increase the plausibility of the changes (Table S3).

### 2.7. Statistical Analysis

Statistical analyses were performed using Student’s *t*-test and/or the one-way ANOVA for microbial reduction analysis. For transcriptome changes, analyses were performed using two-way ANOVA, for groups of genes. For the analyses, *p* ≤ 0.05 was considered significant. The statistical analyses were performed using Prism 8.0.1 (Prism, GraphPad Software, San Diego, CA, USA).

## 3. Results

**Figure 1:**
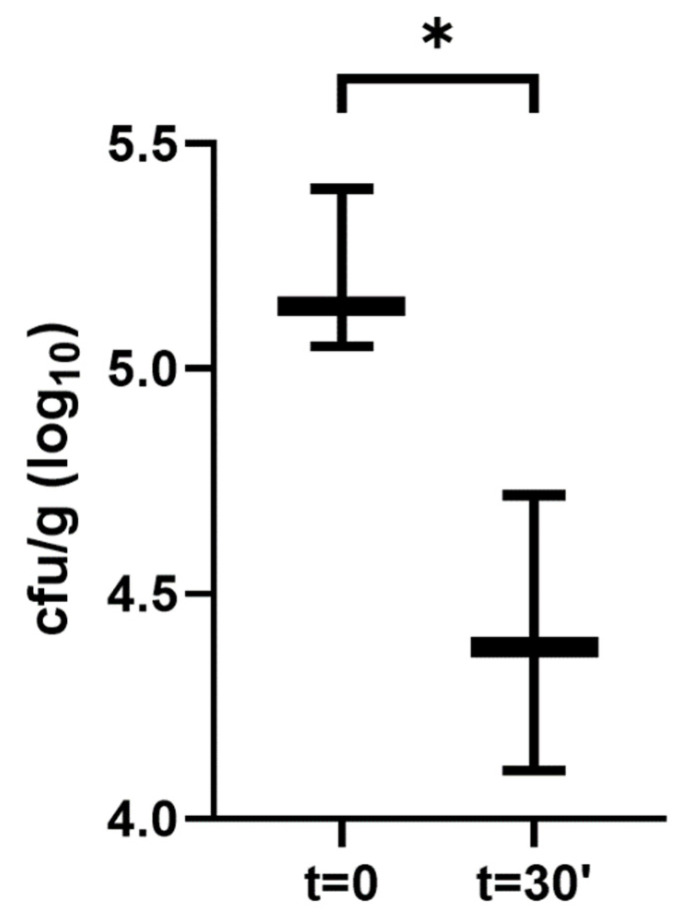
The reduction in viable rate of bacteria during ceviche preparation, *—*p* = 0.0182.

As a result of the conducted experiment, it was observed that during the traditional preparation of ceviche, i.e., marinating for 30 min, a statistically significant (*p* < 0.05) reduction in microorganisms were noticed, although the reduction was from 5.2 to 4.4 log cfu/g (Figure 1).

As a result of the experiment, it was observed that 10 morphologically dominant colonies belonged to four species: *Staphylococcus captis, Staphylococcus epidermidis, Candida parapsilosis* and *Carnobacterium divergens*. The results are shown in Table 1.

**Figure 2:**
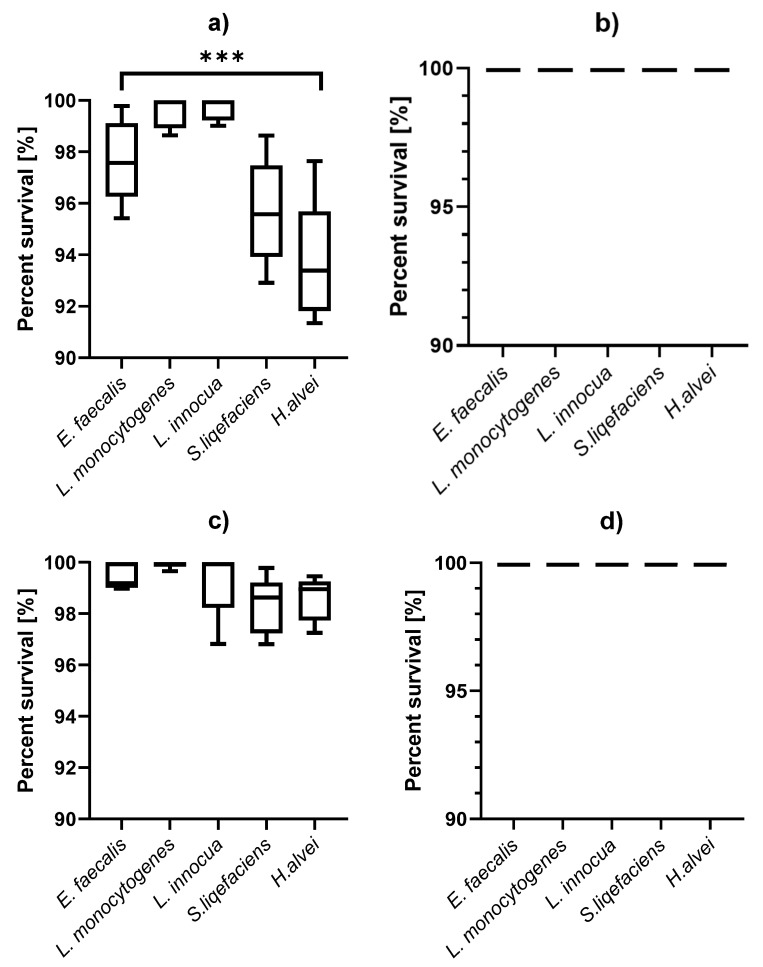
Reduction in the number of live cells of selected species by individual ceviche components: (**a**) lime (23.5%), (**b**) habanero (0.6%), (**c**) onion (5%), (**d**) coriander (3%), ***—*p* < 0.0001.

In the experiment, it was observed that the traditional use of habanero pepper and coriander did not affect the reduction in bacteria from the species *E. faecalis, L. monocytogenes, L. innouca, S. liqefaciens* and *H. alvei* (Figure 2). In the case of the use of onion, it was observed that *L. monocytogenes* was the most resistant and the number of viable cells remained at 99.93% after 30 min. The species *S. liqefaciens* 98.31% were the most sensitive to the use of the onion. Of the four ingredients used, lime juice was the most effective against all bacteria. Again, the most sensitive bacteria were *S. liqefaciens* (95.67% of live cells), while the most resistant ones were *Listeria* sp., 99.57% of live *L. monocytogenes* cells and 99.69% of live *L. innocua* cells.

**Figure 3:**
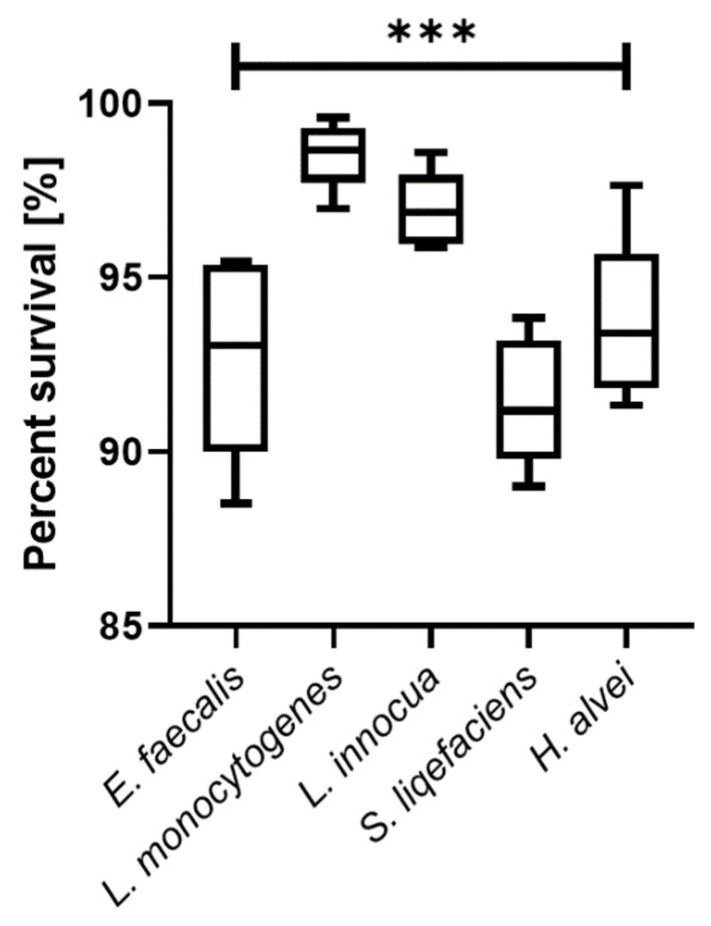
Reduction in the number of live cells of selected species of bacteria during the preparation of ceviche, ***—*p* < 0.0001.

The study of microbial reduction during the preparation of ceviche has proven that the complex action of many components is more effective than the action of individual components (Figure 2 and Figure 3). The highest percentage of viable cells was observed in the case of *L. monocytogenes* and remained at the level of 98.54%, slightly less for *L. innocua* bacteria—96.93%. For the remaining species the reduction did not exceed 10%, for *E. faecalis* it was 92.76%, for *S. liqefaciens* 91.44%, *H. alvei* 93.68%.

**Figure 4:**
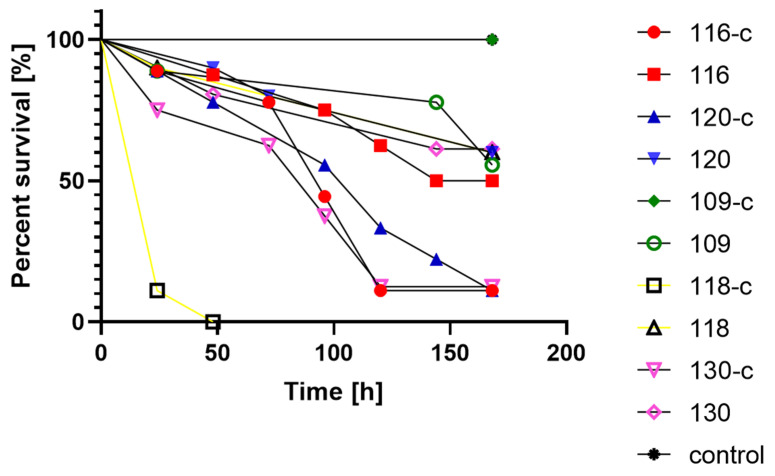
Survival curves of *G. mollonella* larvae injected with *L. monocytogenes*; c—ceviche.

In the conducted experiment on a live model using *Galleria mollonella* larvae, it was observed that the results from the control sample and from the process of preparing the ceviche differ statistically significantly (*p* < 0.05) (Figure 4). Only one strain (109) was more virulent after the ceviche preparation process than before. In the case of the remaining strains, a significant decrease in mortality among larvae was observed. The most drastic change was observed for strain 118, where the death of each infected larva after 48 h was recorded, while 60% of the larvae were still alive after 168 h of onion preparation.

In the conducted study, the expression of selected genes for two serotypes of *L. monocytogenes* 1/2a and 1/2c was tested. The results of the research showed that for the expression of genes from each group, higher results were obtained for serotype 1/2a. Comparing genes related directly or indirectly to virulence, it was observed that significantly overexpression was noticeable for the *inl*A gene, the stress induced by ceviche preparation did not noticeably affect the relative expression of the *fla*A gene responsible for movement. Expression of the stress response genes indicated that the highest expression value, 1.17 for serotype 1/2a and 1.16 for serotype 1/2c, was observed for the *csp*L gene. For the remaining genes: *gad*B, *opuC*A and *rpo*E, in the case of 1/2c, underexpression was observed with the results of 0.93, 0.51 and 0.19, respectively. For the serotype 1/2a, these values were slightly higher and amounted to 1.06 for *gad*B and *rop*E and 0.37 for *opuC*A.

**Figure 5:**
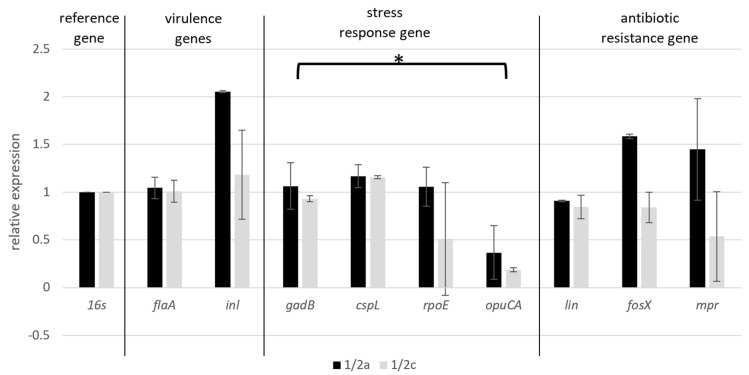
Relative expression of selected *L. monocytogenes* strains, *—*p* = 0.0393.

For the antibiotic resistance genes, it was shown that for the serotype 1/2a, the *fosX* and *mpr* genes were overexpressed during the stress associated with ceviche preparation, while underexpression was observed for the *lin* gene responsible for lincomycin resistance. For serotype 1/2c, underexpression occurred with every gene (Figure 5).

**Figure 6:**
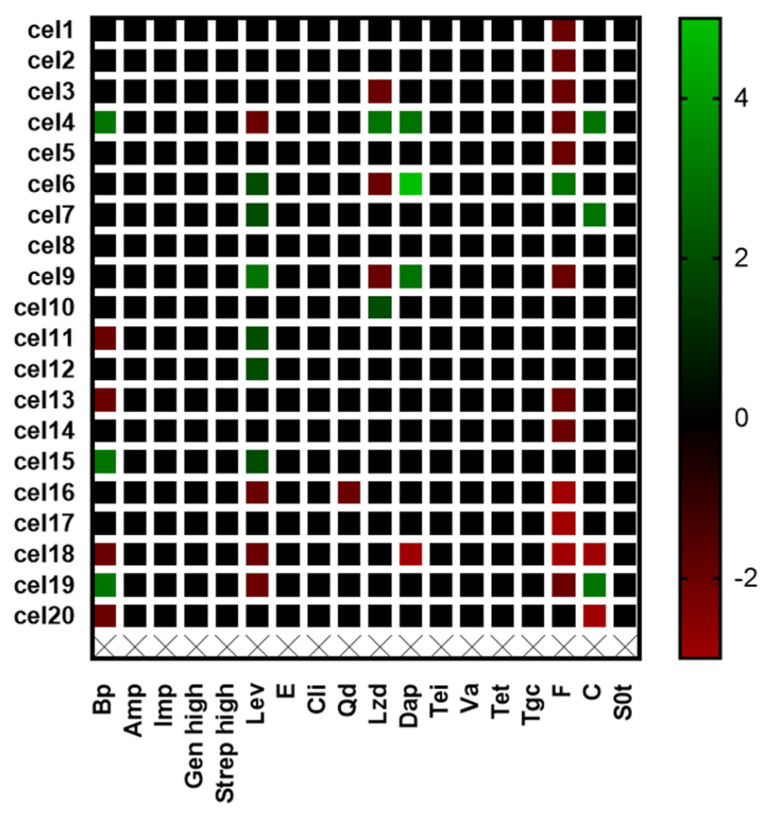
Antibiogram changes after ceviche preparation among *L. monocytogenes* strains; benzypenicillin (Bp), ampicillin (Amp), high-level gentamicin (Gen High), high-level streptomycin (Strep High), clindamycin (Cli), levofloxacin (Lev), erythromycin (E), quinupristin/dalofopine (Qd), linezolid (Lzd), vancomycin (Va), tetracycline (Tet), tigecycline (Tgc), chloramphenicol (C), daptomycin (Dap), Trimethoprim/sulphamethoxazole (Sxt), and nitrofurantoin (F).

As a result of the conducted research, it was shown that out of 18 tested antibiotics, among 11, no changes caused by stress related to the preparation of ceviche were observed (Figure 6). Changes for the largest number of strains were observed in the case of nitrofurantoin, for which the stressor caused a decrease in the MIC value for most of the strains. For levofloxacin, the MIC value increased for as many as six strains, while for four strains it increased. The greatest decrease was observed in the case of nitrofurantoin and chloramphenicol, which, for some whispers, was three times lower than in the case of optimal conditions. In contrast, the greatest increase in MIC values was observed with daptomycin (five-fold increase).

## 4. Discussion

An experiment was carried out to determine the viability of bacteria often isolated from fish—*Listeria* sp., *E. faecalis*, *H. alvei* and *S. liqefaciens*—in the preparation of a traditional Peruvian ceviche. As a result of the experiment, it was proved that despite the combination of several components with the potential antibacterial effect, no significant decrease in viability was observed in any of the tested microorganisms. Although the preparation of ceviche is an effective method to reduce, but not eliminate, bacteria of the genus *Vibrio*, in the case of other microorganisms there is no significant decrease in the number of viable cells [9]. In a study conducted by Mathur et al. on the effects of lime juice, the main antibacterial agent in ceviche, the reduction in Gram-negative bacteria of the genus *Salmonell*a was shown to be as low as 0.5log cfu/g [9]. In our study, although the bacteria of the species *S. liquefaciens* were the most sensitive to the components of ceviche, the reduction was only 10%, it may additionally explain the frequent occurrence of even Gram-negative bacteria in sold RTE ceviche [10].

Identification of the dominant species has shown that *S. captis*, *S.s epidermidis*, *C. parapsilosis* and *C. divergens* are among them. The research results are in line with those obtained by Ramírez-Martínez et al., in which they examined the microbiota of ceviche products; due to the metagenome study usage, researchers were able to assess the microbiome much more accurately [4]. Among the genera overlapping with the researchers is the genus *Staphylococcu*s belonging to the phylum *Firmicutes,* although the researchers did not specify the species. Additionally, *C. divergens* belongs to the same *Firmicutes* phylium. Eukaryotic types have not been determined.

Aromatic plants and spices such as Umbelliferae/Umbelliferae coriander (*C. sativum* L.) contain biologically active compounds with antibacterial, antifungal and antioxidant properties and are widely used in the pharmaceutical and food processing industries. Coriander is a bare aromatic herbaceous annual plant with a long culinary history and serves as a source of fragrances and essential oils, and those are usually considered as affective against variety of microorganisms.

In research conducted by Kačániová et al. [11] it was shown that the main volatile compounds of coriander essential oil were β-linalool 66.07%, camphor tree 8.34%, geranyl acetate 6.91% and cymene 6.35% and expressed the strongest antibacterial activity against *B. subtilis* followed by *S. maltophilia* and *P. expansum.* However, research on coriander extracts showed that it has poor or non-antimicrobial properties [12,13,14], which was also proven in our research, although a more sensitive method of antimicrobial activity was performed.

As in the case of aromatic plants, habanero peppers also have an antibacterial potential, while capsaicin and its derivatives are characterized by high MIC values of 512 mg/L against most Gram-negative bacteria, including *Escherichia coli, Pseudomonas aeruginosa, Klebsiella pneumoniae,* and *Proteus* species and shows a slightly lower MIC value for Gram-positive including various methicillin-resistant bacteria. A minimum MIC of 128 mg/L was determined for capsaicin against *Bacillus subtilis* [15]. Although habanero pepper extracts may contain as much as 5.88 mg/mL of capsaicin, its amount and very short exposure time do not affect the viability of bacterial cells.

While testing the individual components of ceviche, onion resulted in a slight reduction in the number of viable cells, ranging from 98.31% for *S. liqefaciens* to 99.93% for *L. monocytogenes*. There are numerous reports of the antibacterial effects of onions. Ramos et al. [16] examined onion waste containing dried onions as the main component and excellent antibacterial and antioxidant properties were observed. In addition, high antibacterial activity of onion skin waste extract against *E. coli, P. fluorescens, B. cereus*, and the fungi *Aspergillus niger, Trichoderma viride*, *Penicillium cyclopium* was observed [17]. It has been reported that onion excretion contains a large amount of phenol and flavonoids, mainly quercetin, which has high antibacterial activity [18].

In our experiment, it was determined whether the preparation of ceviche can influence the virulence expressed phenotypically in the model using *G. mollonella* larvae, and the results showed that only one *L. monocytogenes* strain was observed to be more virulent after preparing the ceviche than in the control. This can be explained by sudden and short-term stress, as there are reports explaining that the greatest changes in the transcriptome of genes responsible for virulence are observed after prolonged exposure to the stress factor which is lowering the pH. [19,20]. Li et al., using *G. mollonella* larvae to study the virulence of *L. monocytogenes* exposed to organic acids, proved that the action of the stress factor causes an increase in the pathogenic potential for a long time; however, those are still strain-dependent features [20,21,22].

The main stressor in the preparation of ceviche is the lowered pH. This stress contributes to the upregulated gene expression associated with acid stress. In the case of *L. monocytogenes*, several decarboxylation systems are responsible for survival [23]. These decarboxylation systems include the glutamate decarboxylase (GAD) and the arginine decarboxylase (ADI) systems. The GAD system comprises three homologous glutamate decarboxylases, GadD1, GadD2, and GadD3 and two antiporters GadT1 and GadT2, encoded at three distinct genetic loci [24,25]. In this study, the expression of one of the genes encoding GAD was investigated and it was observed that overexpression occurs only in serotype 1/2a, while resistance to low pH in serotype 1/2c may be related to the ADI system, which consists of three enzymes and one membrane bound transporter: arginine deiminase (ArcA), catabolic ornithine carbamoyltransferase (ArcB, also known as AguB), carbamate kinase (ArcC, also known as AguC), and the arginine/ornithine antiporter ArcD [26]. The action of stressors may also influence the expression of other genes, including virulence genes. There is ample evidence that high osmolarity, bile, acid, ethanol, blood increase the virulence potential of *L. monocytogenes* strains [26]. The study examined the effect of lowering the pH during the preparation of ceviche on the *inl* and *fla*A genes and observed significant expression induction for serotype 1/2a.

*L. monocytogenes* strains are generally susceptible to a wide range of antibiotics, occasionally antibiotic resistant *L. monocytogenes* strains have been observed. The first multidrug-resistant strain of *L. monocytogenes* was reported in 1988 by Poyart-Salmeron et al. (1990) [27]. This is confirmed by the characteristics of the *L. monocytogenes* strains used in our study, which were mainly resistant to two antibiotics, daptomycin and nitrofurnantoin. The stress of ceviche preparation resulted in changes, especially with nitrofurantoin and chlorpahonicol, where a decrease in MIC was observed, and doptamycin, with the greatest increase. In a study of the impact of stress associated with the food industry and cooking, Al-Nabulsi et al. only observed a positive trend, an increase in MIC values during acid stress [28,29]. Results obtained by McMahon et al. showed that NaCl or acid stress on *E. coli* and *S. aureus* cells were more resistant (4-fold) to the antibiotics tested than control cells. On the other hand, *S. Typhimurium* cells stressed after NaCl or acid stress were comparable to or less resistant to unstressed cells. The differences in the research may result from the use of *L. monocytogenes* by the authors of the stocks, after a minimum of 1 day after stress (about 10 generations of cells). These can vary considerably between stalks and serogroups. The changed antibiotic resistance observed in this study is possibly linked to the reduction in cell wall antibiotic binding sites, the amplification of genes involved in the synthesis and manipulation of excretion pumps, and the induction of stress shock proteins [30].

## 5. Conclusions

The conducted study allowed us to determine that despite the fact that almost all of the components in ceviche preparation have bactericidal potential, the use of fresh ingredients and a short exposure time to these ingredients is not effective in reducing *L. monocytogenes* and other bacteria that are commonly isolated from fish meat. Therefore, it is important to ensure the quality and accurate pretreatment of the products used to prepare the ceviche, as the stress associated with bacteria may contribute to negative changes of the strains.

## Figures and Tables

**Table 1:** Identification of the most common species after the preparation of ceviche using MALDI-TOF technique.

No.	Species	Identification to Species Level%	Quality of Protein Extraction (Datacount)
1	*S. captis*	91.80–99.90	117–182
2	*S. epidermidis*	99.00	130–158
3	*C. parapsilosis*	99.00	117–132
4	*C. divergens*	75.50–92.00	144–152

## Data Availability

The dataset used during this study is the available form given author upon reasonable request.

## Supplementary Materials

The following are available online at https://www.mdpi.com/article/10.3390/foods11060860/s1, Table S1: Strains used in study, Table S2: Antibiogram of L, monocytogenes strains used in study, Table S3: RT-qPCR oligonucleotide primers used in this study.
